# 2945. Audit and Feedback Informed ASP Interventions Lead to Sustained Reductions in Suboptimal Antibiotic Prescribing at Hospital Discharge

**DOI:** 10.1093/ofid/ofad500.184

**Published:** 2023-11-27

**Authors:** Lauren M Puckett, Laura Bio, Sean Cornell, Hayden T Schwenk

**Affiliations:** Stanford Children's Hospital, Stanford, CA; Lucile Packard Children's Hospital Stanford, Palo Alto, CA; Lucile Packard Children's Hospital Stanford, Palo Alto, CA; Stanford University School of Medicine, Stanford, California

## Abstract

**Background:**

Antimicrobial stewardship programs (ASPs) have traditionally focused on the inpatient setting; however, a significant proportion of antibiotics initiated during hospital stays are continued at the time of discharge. Previous studies have found that rates of suboptimal antibiotic prescribing at the time of hospital discharge may be as high as 30%. Our study aimed to implement ASP interventions targeted at reducing suboptimal prescribing at the time of hospital discharge and to assess the effectiveness and durability of these interventions over time.

**Methods:**

Beginning 11/2020, our ASP began audit and feedback of both enteral and parenteral hospital discharge antibiotic prescriptions. A prescription was determined to be suboptimal if the dose, frequency, duration, selection, or formulation were inconsistent with institutional and/or national guidelines or based on the clinical judgment of the ASP pharmacist. Findings from this process were used to develop interventions, including targeted provider and medical service education, institutional guideline development, and electronic health record modifications. The rate of suboptimal discharge antibiotic prescribing from 12/2020-4/2023 was plotted on a statistical process p-chart, including a historical baseline (9/2020-10/2020).

**Results:**

A total of 4347 prescription audits were performed over a 28-month period and included 4211 enteral and 136 parenteral antibiotics. Reasons prescriptions were identified as suboptimal are included in Figure 1. A p-chart (Figure 2) identified a decline in suboptimal prescribing from a baseline rate of 29% to 15% after implementation of audit and feedback, modification of medication order entry panels, and targeted interventions to surgical services. After implementation of all interventions (Table 1), a centerline shift was detected, with a decrease in suboptimal prescribing from 15% to 8%.

Figure 1.

**Figure:**
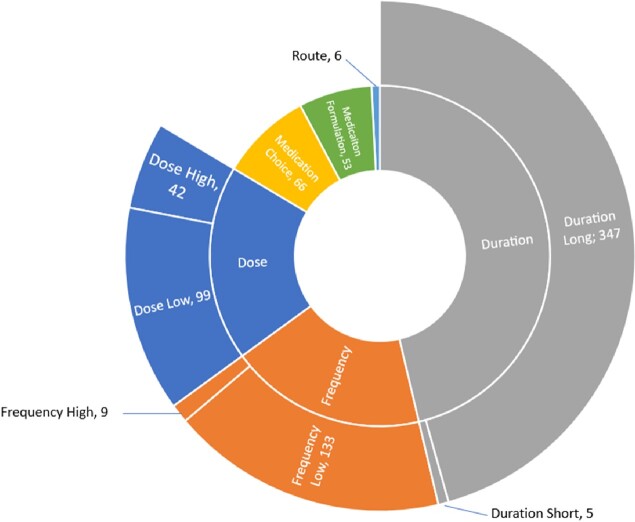

Reasons discharge prescriptions were identified as suboptimal (N=568). A prescription may have more than one reason for being considered suboptimal.

Figure 2.

**Figure:**
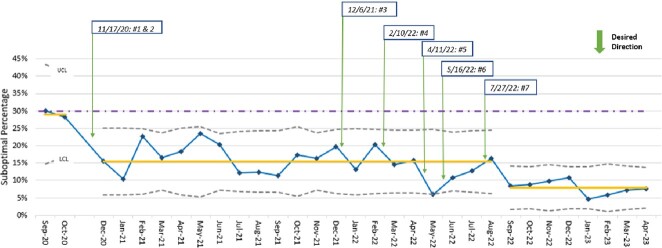

Suboptimal Antibiotic Prescriptions at Hospital Discharge, 2020 – 2023: P-chart for suboptimal prescription rate. The yellow line indicates mean suboptimal prescription percentage for the study period (15% and 8%, respectively). The purple dashed line indicates literature reported rate of suboptimal prescribing (30%). Interventions annotated 1-7 within figure are found in Table 1. LCL, lower control limit; UCL, upper control limit.

**Figure:**
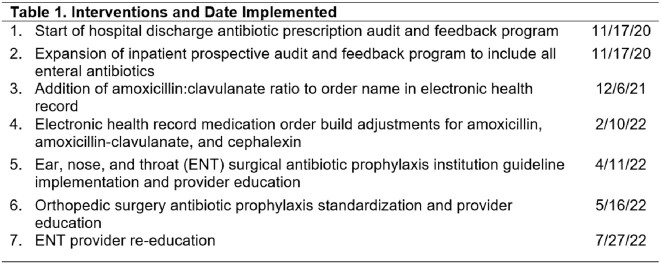

**Conclusion:**

Suboptimal antibiotic prescribing rates decreased by 72% over a 2.5-year period through a mix of audit with feedback and targeted interventions. These new processes may demonstrate a potentially effective sustained reduction in suboptimal prescribing. ASPs should consider implementation of hospital discharge prescription review as part of ambulatory stewardship efforts.

**Disclosures:**

**All Authors**: No reported disclosures

